# The human box C/D snoRNAs U3 and U8 are required for pre-rRNA processing and tumorigenesis

**DOI:** 10.18632/oncotarget.11148

**Published:** 2016-08-09

**Authors:** Jean-Louis Langhendries, Emilien Nicolas, Gilles Doumont, Serge Goldman, Denis L.J. Lafontaine

**Affiliations:** ^1^ RNA Molecular Biology, Fonds de la Recherche Scientifique (F.R.S.-FNRS), Université Libre de Bruxelles (ULB), BioPark Campus, Gosselies, Belgium; ^2^ Nuclear Medecine, Erasme Hospital, Université Libre de Bruxelles, Belgium; ^3^ Center for Microscopy and Molecular Imaging (CMMI), BioPark campus, Université Libre de Bruxelles, Belgium

**Keywords:** snoRNA, nucleolus, ribosome, tumorigenesis, cancer

## Abstract

Small nucleolar RNAs (snoRNAs) are emerging as a novel class of proto-oncogenes and tumor suppressors; their involvement in tumorigenesis remains unclear. The box C/D snoRNAs U3 and U8 are upregulated in breast cancers. Here we characterize the function of human U3 and U8 in ribosome biogenesis, nucleolar structure, and tumorigenesis. We show in breast (MCF-7) and lung (H1944) cancer cells that U3 and U8 are required for pre-rRNA processing reactions leading, respectively, to synthesis of the small and large ribosomal subunits. U3 or U8 depletion triggers a remarkably potent p53-dependent anti-tumor stress response involving the ribosomal proteins uL5 (RPL11) and uL18 (RPL5). Interestingly, the nucleolar structure is more sensitive to perturbations in lung cancer than in breast cancer cells. We reveal in a mouse xenograft model that the tumorigenic potential of cancer cells is reduced in the case of U3 suppression and totally abolished upon U8 depletion. Tumors derived from U3-knockdown cells displayed markedly lower metabolic volume and activity than tumors derived from aggressive control cancer cells. Unexpectedly, metabolic tracer uptake by U3-suppressed tumors appeared more heterogeneous, indicating distinctive tumor growth properties that may reflect non-conventional regulatory functions of U3 (or fragments derived from it) in mRNA metabolism.

## INTRODUCTION

Ribosomes are cellular nanomachines essential for protein production in all living cells. In eukaryotes, ribosome biogenesis is initiated in the nucleolus, a specialized subcompartment of the cell nucleus where ribosomal RNA (rRNA) precursors are synthesized by RNA polymerase I [1]. In addition to being extensively processed, i.e. undergoing multiple cleavages that ultimately yield the mature rRNAs (Figure S1 and [2, 3]), precursor rRNAs (pre-rRNAs) are heavily modified post-transcriptionally [4]. This can involve isomerization of specific nucleobases (conversion of uridines to pseudouridines) or addition of particular chemical groups to specific nucleotides (i.e. acetylation, aminocarboxylpropylation, and methylation, see [5]). rRNA modifications are assumed to optimize ribosome function, although in most cases this remains largely hypothetical [5].

Small nucleolar RNAs (snoRNAs) are abundant small stable RNAs that localize to the nucleolus, where they are involved in pre-rRNA modification and processing [6]. Identified roles include i) contributing to RNA folding into productive conformations (through extensive and sometimes intricate Watson-Crick base-pairing) and ii) recruiting catalytically active proteins to sites of modification or cleavage [7]. On the basis of the presence of conserved primary sequence elements (so-called “boxes”), conserved secondary folds, and association with specific proteins, three classes of snoRNAs have been defined: the box C/D, box H/ACA, and MRP (mitochondrial RNA processing) snoRNAs [7]. The vast majority of box C/D and box H/ACA snoRNAs are active in modification (respectively in 2′-*O* methylation and pseudouridylation), but a few members of each of these families are involved in processing. Among these are the box C/D snoRNAs U3 and U8, which are the subject of this work. In budding yeast, the RNase MRP is involved in processing nucleolar pre-rRNAs [8], a function that surprisingly might not have been conserved in human cells [9]. This observation highlights the need to explore in detail the functions of snoRNAs in different organisms, even in cases of structural conservation, to avoid fallacious extrapolations.

U3 is present in all eukaryotes inspected to date, while U8 has been reported only in vertebrates [10–16]. The processing functions of U3 and U8 have been documented in multiple experimental models, but surprisingly not in human. In yeast [11, 17, 18], frog [12, 19–21] and mouse [13], U3 is required for processing reactions leading to synthesis of the small subunit rRNA. On the other hand, work conducted in frog [15, 22–24] and mouse [25] has demonstrated that U8 is required for cleavage reactions leading to synthesis of large subunit rRNAs.

Although the general architecture of the pre-rRNA processing pathways is well conserved across eukaryotes [2, 3], there are important species-specific differences. These include additional *trans*-acting factors, the existence of redundant maturation pathways, additional cleavage sites, and significant differences in the order of cleavages [26, 27]. Collectively, these differences make it absolutely essential to establish the precise processing functions of U3 and U8 in human cells. This is relevant notably to our understanding of ribosomopathies, which are cancer predisposition syndromes caused by ribosome biogenesis dysfunction [28, 29]. The aim of this work was to characterize the functions of U3 and U8 in ribosome biogenesis in human cells.

U3 and U8 are potential cancer biomarkers. Elevated levels of these snoRNAs are consistently observed in human breast cancers [30]. Recent research has further shown that snoRNA genes can act as proto-oncogenes or as tumor-suppressors, that regulation of their expression is altered in cancer, and that this contributes to cell transformation, tumorigenesis, and metastasis [31–33]. Case reports describe the involvement of both box C/D (SNORD) and box H/ACA (SNORA) snoRNAs in brain, breast, cervical, liver, lung, and prostate cancers [31–33]. For example, the genes encoding SNORA42 and SNORD78 act as proto-oncogenes in non-small-cell lung cancer [34, 35], while SNORD76 acts as a tumor suppressor in glioblastoma [36] and U50 as a tumor suppressor in prostate and breast cancers [37, 38]. Several core snoRNP proteins, such as fibrillarin, and snoRNP assembly factors, have also been linked to cancer [30, 39, 40]. With a few notable exceptions, such as SNORD50A and SNORD50B, which inhibit K-Ras through direct binding [41], it remains totally unclear how snoRNAs contribute to tumorigenesis. Given their tumorigenic potential, snoRNAs are emerging as a novel class of cancer biomarkers [31]. They are readily detectable in body fluids such as blood plasma and serum, and hold great promise as circulating biomarkers for disease diagnosis and prognosis [31].

Antisense interfering oligonucleotides are stable in blood. Their recently demonstrated ability to silence *in vivo* non-coding RNAs, and notably the melanoma-specific lincRNA *SAMMSON* [42], indicates potential applications for these oligonucleotides in cancer therapy. In a recent study, Su *et al.* [30] globally depleted an entire family of several hundred small nucleolar RNAs by targeting shared protein components essential to their metabolic stability. This led to reduced tumorigenicity of cancer cells both *in vitro* and *in vivo* [30]. We reasoned that targeting a single snoRNA molecule such as U3 or U8 and clearly establishing its molecular function in human cells might prove to be a more powerful and specific means of achieving therapeutic goals than targeting an entire class of snoRNAs.

In this work, we have investigated the involvement of the human box C/D snoRNAs U3 and U8 in ribosome biogenesis and tumorigenesis.

## RESULTS

### The box C/D snoRNAs U3 and U8 are required for pre-rRNA processing in human cells

To investigate the function of U3 and U8 in human ribosome biogenesis, specific anti-sense oligonucleotides (ASOs) were used to deplete lung (H1944) and breast (MCF-7) cancer cells of these snoRNAs. To monitor the time course of snoRNA depletion, total RNA was extracted after 24, 48 and 72 hours of treatment. The efficiency of snoRNA depletion was confirmed by Northern blotting, RT-qPCR, and growth monitoring (Figure S2).

**Figure 1:**
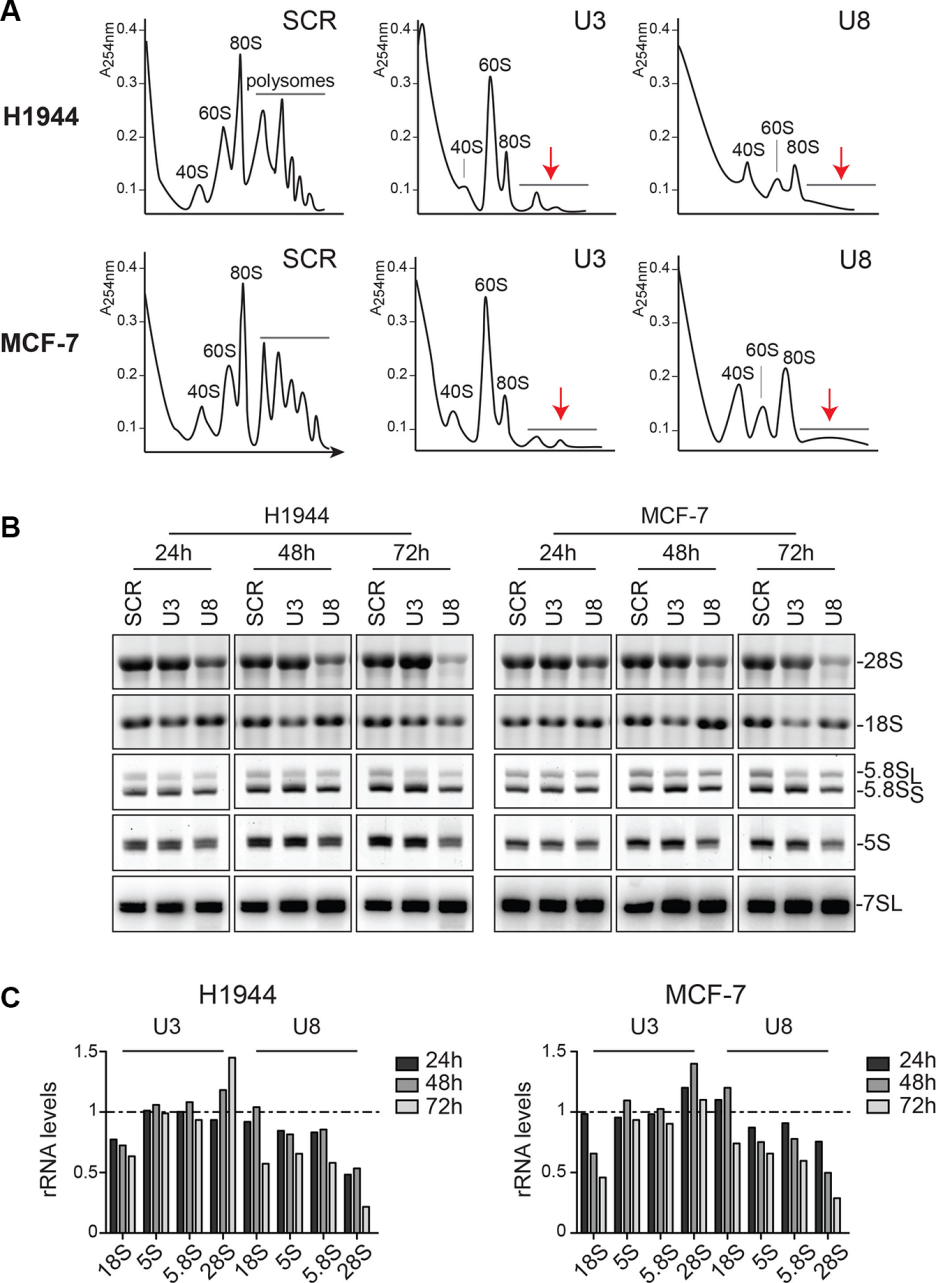
The box C/D snoRNAs U3 and U8 are required for human ribosomal subunit biogenesis (**A**) Polysomes analysis. H1944 and MCF-7 cells were depleted of U3 or U8 for two days and then treated with cycloheximide, to freeze the polysomes. Total extracts were analyzed by sucrose gradient centrifugation. The reduction of polysomes upon snoRNA depletion is obvious (red arrows). U3 depletion leads to a marked subunit imbalance due to a deficit in 40S, while U8 depletion affects primarily 60S accumulation. (**B**) Mature rRNA accumulation. H1944 and MCF-7 cells were depleted of U3 or U8, for 1, 2, or 3 days. Total RNA was extracted, resolved on denaturing gels, and mature rRNAs were visualized by ethidium bromide staining. 7SL, detected by Northern blotting, was used as a loading control. (**C**) Densitometric quantification of the signals shown in panel B. rRNA levels in cells depleted for U3 or U8 normalized with respect to the levels observed in cells treated with a non-targeting silencer (SCR). Note that after 72 h of U8 depletion, in addition to inhibition of 28S rRNA synthesis, the 18S and other rRNAs, were also reduced; this reflects the important reduction in cell viability observed at this time-point (Figure S2D).

As an initial readout for characterizing the function of U3 and U8 in human ribosome synthesis, we established polysome profiles in H1944 and MCF-7 cells depleted of one of these snoRNAs for two days (Figure 1A). Cells treated with a non-targeting control silencer (SCR) showed the characteristic peaks corresponding to the small and large subunits (40S and 60S, respectively), monosomes (80S), and polysomes. In cells depleted of U3 or U8, the amount of polysomes was drastically reduced. In the case of U3 depletion, 40S accumulation was the most strongly affected, while U8 depletion had a major impact on 60S accumulation (Figure 1A). Consequently, the amounts of 80S were reduced in both cases.

Total RNA was resolved on denaturing gels and steady-state levels of mature rRNAs were established by ethidium bromide staining (for 5S, 5.8S, 18S, and 28S) or Northern blotting (for the 7SL loading control)(Figure 1B). Densitometric quantification confirmed that U3 depletion leads to a reduction in 18S, while U8 depletion mostly affects 28S synthesis (Figure 1C). This is consistent with the observed changes in polysome profiles (Figure 1A).

**Figure 2:**
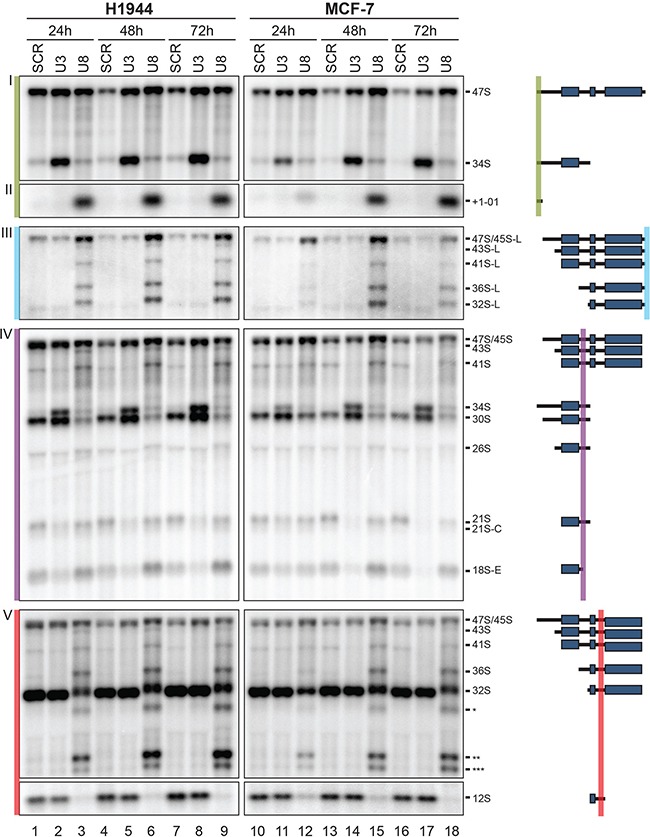
U3 and U8 are required for pre-rRNA processing in human cells Total RNA extracted from H1944 or MCF-7 cells depleted of U3 or U8 for 1, 2, or 3 days was resolved on denaturing gels and analyzed by Northern blotting with specific probes (see Materials and Methods). As a control, cells were treated with a non-targeting silencer (SCR). Blots were probed with oligonucleotide LD1844 (panels I and II), LD2612 (panel III), LD1827 (panel IV), and LD1828 (panel V). The pre-rRNA species detected are indicated and represented as schematics with the probes used highlighted. A detailed pre-rRNA processing pathway and a description of all the RNA species detected are provided in Figure S1. Note that several abundant truncated forms of the 36S RNA are detected (highlighted with stars in panel V). We suggest that in the absence of ITS2/3′-ETS processing after U8 depletion, large subunit precursors are targeted for degradation, and that this is manifested by activation of cryptic cleavage sites within the 28S-coding part of the precursor, leading to production of these abnormal dead-end intermediates.

The 18S, 5.8S, and 28S rRNAs are encoded in a single transcript synthesized by RNA polymerase I. In this precursor, they are embedded between non-coding spacer sequences, which include the 5′ and 3′ external transcribed spacers (ETS) and the internal transcribed spacers (ITS) 1 and 2 (Figure S1A). The 5′ and 3′ termini of mature rRNAs are produced through extensive processing (Figure S1 and ref. [2]). A detailed pre-rRNA processing analysis was performed by Northern blotting with specific probes designed to detect all major pre-rRNA intermediates (Figure 2, Figures S1 and S3, see ref. [26]). In agreement with the effects reported on ribosomal subunit accumulation in the polysome analysis, U3 and U8 appeared essential to RNA cleavage steps leading, respectively, to production of the small- and large-subunit rRNAs (Figures 2 and S3).

U3 depletion was found to affect processing in the 5′-ETS and ITS1, as shown by inhibition of cleavages at sites 01 and A0, leading to accumulation of the aberrant 34S RNA (Figure 2, panels I and IV and Figure S3), and by the absence of cleavage at sites 2, C, and E, with concomitant loss of the 21S, 21S-C, and 18S-E pre-rRNAs (Figure 2, panel IV, and Figure S3). In agreement with the view that cleavages in the 5′-ETS are inhibited upon U3 depletion, clear accumulation of the 47S primary transcript was observed at the 48 h and 72 h time points (Figure 2 and Figure S3).

In contrast, U8 depletion was found to inhibit processing primarily in ITS2 and the 3′-ETS. The absence of processing in the 3′-ETS was evidenced by accumulation of multiple pre-rRNAs having retained 3′-ETS sequences and showing abnormal extension in 3′ (45S-L, 43S-L, 41S-L, 36S-L and 32S-L, Figure 2, panel III and Figure S3). Inhibition of maturation in ITS2 led to a marked reduction in 32S, the total disappearance of 12S (Figure 2, panel V and Figure S3), and a reduction in 5.8S+40 (Figure S4). In addition, U8 depletion also affected processing in the 5′-ETS and within ITS1. Inhibition of cleavages in 5′-ETS was substantiated by accumulation of 47S (Figure 2, panel I and Figure S3) and of the +1-01 fragment (Figure 2, panel II and Figure S3). Inhibition of processing within ITS1 was demonstrated by production of the aberrant 36S RNA (Figure 2, panel V and Figure S3) and concomitant loss of 30S (inhibition of cleavage at site 2, Figure 2, panel IV and Figure S3). The +1-01 spacer fragment is a portion of the 5′-ETS that is normally turned over by the 5′-3′ exoRNase XRN2 [43]. The observed accumulation of this +1-01 RNA suggests the existence of functional interactions between early- and late-acting processing complexes, i.e. between processing factors involved in 5′-ETS and ITS2/3′-ETS maturation.

The effects of snoRNA depletion on processing appeared to be practically the same in H1944 and MCF-7 cells (Figures 2, S3–S4). To see how general this conclusion might be, we carried out U3 or U8 depletion in several additional cell lines: three lung cancer cell lines (H1975, A549, DMS-53), one breast cancer cell line (BT-549), one cervical cancer cell line (HeLa), and a pair of isogenic colon carcinoma cell lines (HCT116 p53^+/+^ and HCT116 p53^−/−^). We found U3 or U8 depletion to affect all these cells quite similarly (data not shown). Use of HCT116 cells expressing p53 or not in an otherwise isogenic background [44], allowed us to further conclude that the involvement of U3 and U8 in processing does not require the presence of p53 (data not shown).

### A p53-dependent antitumor nucleolar surveillance pathway is activated upon depletion of the box C/D snoRNAs U3 or U8

In unperturbed cells, the antitumor protein p53 is maintained at a low level by constitutive polyubiquitination by the E3 ubiquitin ligase Hdm2, followed by proteasomal degradation [45]. When cells undergo a ribotoxic stress, i.e. when ribosome biogenesis is dysfunctional, a nucleolar surveillance pathway is activated which leads to p53 stabilization, cell cycle arrest, apoptosis and cell death [26, 46, 47]. This was found to occur, for example, upon global inactivation of all box C/D snoRNAs after depletion of a shared protein important for their metabolic stability [30]. The question was whether it would also occur after depletion of a single snoRNA.

**Figure 3:**
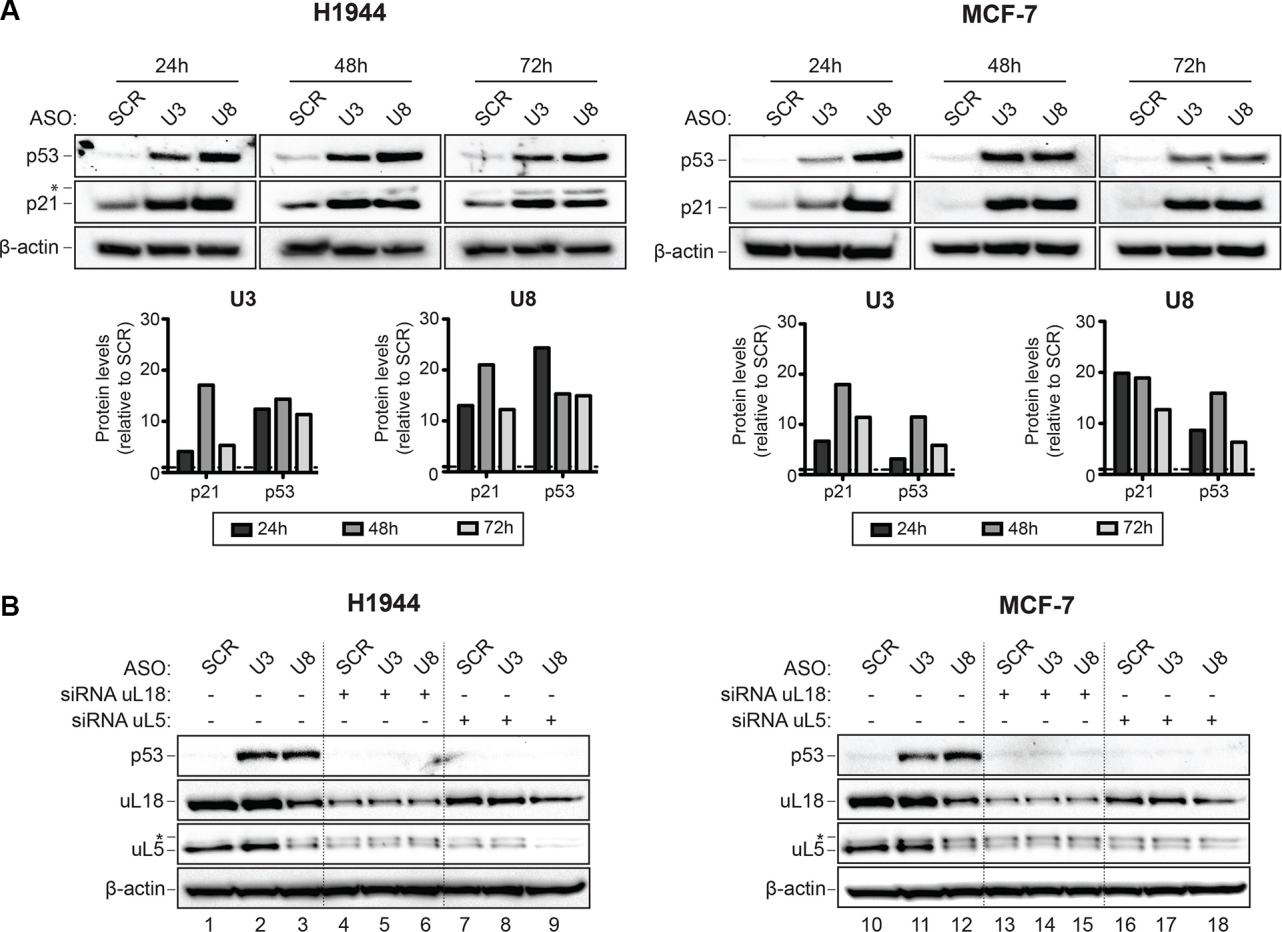
The depletion of U3 or U8 elicits a p53-dependent nucleolar antitumor surveillance pathway (A) U3 and U8 depletion lead to an increased p53 steady-state level. Total protein extracted from H1944 or MCF-7 cells depleted of U3 or U8 for 1, 2, or 3 days was resolved on SDS-polyacrylamide gels and analyzed by Western blotting with specific antibodies (see Materials and Methods). As a control, cells were treated with a non-targeting silencer (SCR). In H1944 cells, at the late time points of depletion (48 and 72 h), p21 was detected as a doublet, suggesting it is post-translationally modified. The signals were quantitated with a ChemiDoc and normalized with respect to SCR-treated cells. As loading control, we probed the blots for β-actin. (B) The increase in p53 steady-state level observed upon U3 or U8 depletion depends on the presence of uL5 and uL18. H1944 and MCF-7 cells depleted of U3 or U8, or treated with a non-targeting control silencer (SCR), were codepleted of uL5 or uL18. Total protein was extracted and analyzed as in panel A. uL5 appears as a doublet, suggesting it is post-translationally modified.

To test whether depletion of U3 or U8 triggers a p53-dependent nucleolar stress response, total protein was extracted from H1944 and MCF-7 cells depleted of U3 or U8 for 3 days and analyzed by Western blotting (Figure 3A). This analysis revealed, in cells depleted of U3 or U8, a substantial (> 10-fold) increase in p53 and in one of its transcriptional targets, p21 (Figure 3A). In H1944 cells, after only 24 hours of U8 depletion, the level of p53 was increased 25-fold. The extent of p53 stabilization observed upon depletion of U3 or U8 was greater than observed after depletion of ribosomal proteins [48].

The current model of nucleolar stress posits that ribosome biogenesis dysfunction leads to the accumulation of unassembled ribosomal components. These include a trimeric 5S RNP particle, consisting of the 5S rRNA and the ribosomal proteins (r-proteins) uL5 (formerly RPL11) and uL18 (RPL5), which sequester Hdm2 and thus prevent it from modifying p53 [49, 50]. As a result, p53 is stabilized. In agreement with this model, we found the p53 steady-state level increase observed upon U3 or U8 depletion to depend on the presence of normal amounts of uL5 and uL18: in cells codepleted of either of uL5 or uL18 and either U3 or U8, p53 was not stabilized (Figure 3B, compare lanes 4–9 and 13–18 with lanes 2–3 and 11–12, respectively, and see Figure S5 for quantitation).

Incidentally, we found U8, but strikingly not U3, to be required for normal steady-state accumulation of uL5 and uL18 (Figure 3B, compare lane 3 with lanes 1 and 2, and lane 12 with lanes 10 and 11; see also Figure S5). We also found uL18 to be required for the metabolic stability of uL5 (Figure 3B, compare lanes 4–6 with lanes 1–2, and lanes 13–15 with lanes 10–11; see also Figure S5). Reciprocally, uL5 appeared to be needed for normal accumulation of uL18 (Figure 3B, compare lanes 7–9 with 1–2, and lanes 16–18 with lanes 10–11; see also Figure S5). The effects of depleting cells of a snoRNA, in combination or not with depletion of an r-protein, were virtually the same in H1944 and MCF-7 cells.

**Figure 4:**
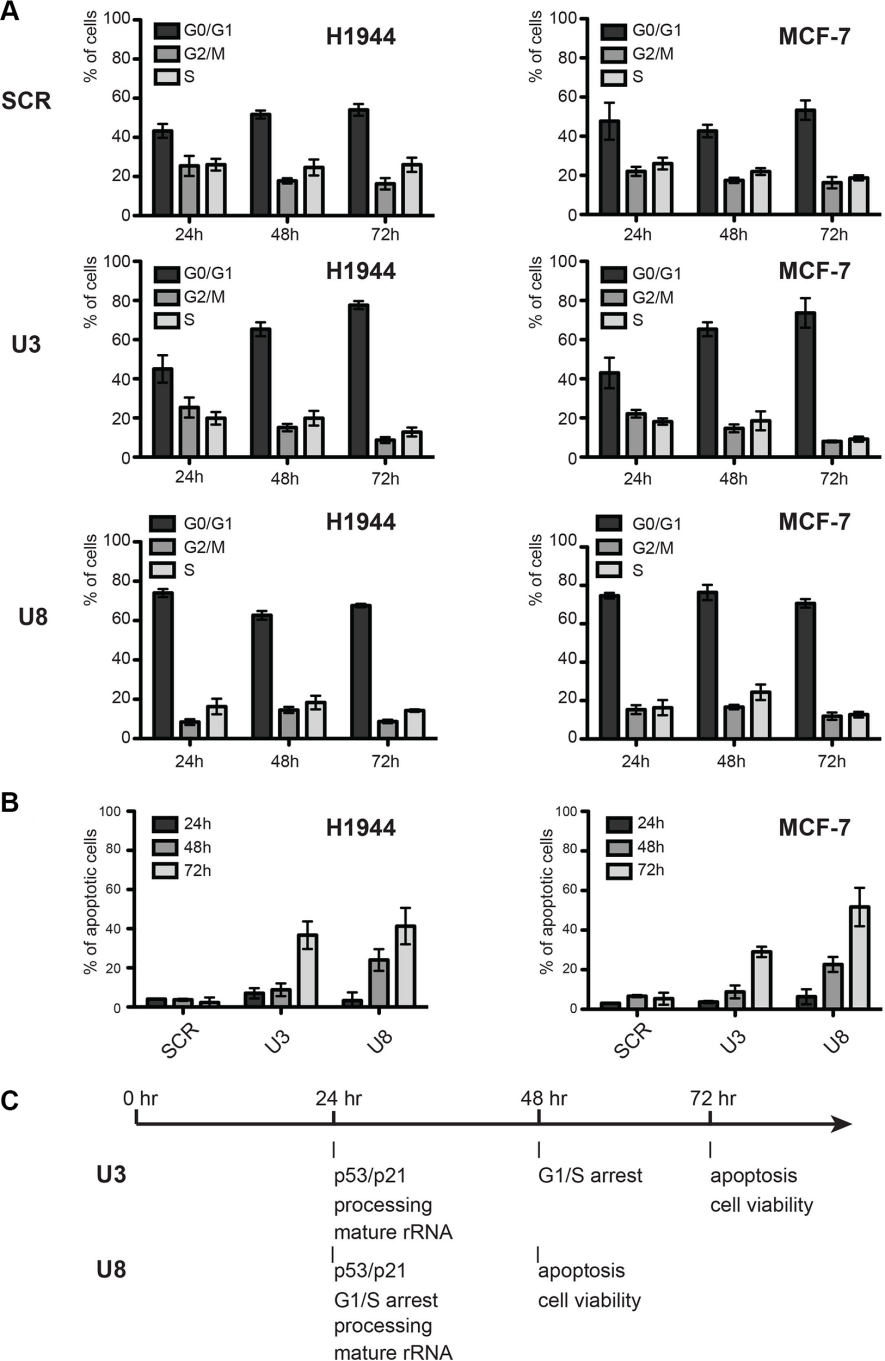
Effects of U3 and U8 depletion on cell-cycle progression and apoptosis (**A**) Cell-cycle analysis. The fractions of cells in the different phases of the cell-cycle (G0/G1, G2/M, or S) in populations of cells depleted of U3 or U8 for 1, 2, or 3 days were determined by staining nuclear DNA with propidium iodide and counting the fluorescence with a Muse (see Materials and Methods). As a control, cells were treated with a non-targeting silencer (SCR). (**B**) Apoptosis analysis. The fraction of apoptotic cells in populations of cells depleted of U3 or U8 or treated with the non-targeting control silencer (SCR) for 1, 2, or 3 days was determined by performing an Annexin V assay and counting the fluorescence with a Muse (see Materials and Methods). (**C**) Timeline of the appearance of phenotypes.

The remarkable accumulation of p53 upon U3 and U8 depletion prompted us to test cell cycle progression and apoptosis. Cell-cycle analysis revealed an important blockage in G1. Upon U3 depletion, the proportion of cells blocked in G1 increased steadily from 40–50% to ∼80% (Figure 4A). In the case of U8 depletion, the effects on cell cycle progression were more acute, reaching a plateau of ∼70% of cells in G1 within 24 hours of the onset of depletion. The proportion of apoptotic cells, measured with an annexin V assay, increased steadily upon U3 or U8 depletion, culminating at > 40% after 3 days (Figure 4B). Overall, the effects on cell cycle progression and apoptosis were quite similar in H1944 and MCF-7 cells.

In conclusion, depletion of U3 or U8 causes inhibition of pre-rRNA processing at specific sites, resulting in reduced production of mature rRNAs and failure to assemble the small (U3 depletion) or large (U8 depletion) subunit. These ribosome biogenesis dysfunctions trigger an antitumor nucleolar stress response leading to an increased steady-state level of p53 (and of p21), prompting cell cycle arrest, apoptosis and cell death. The timing of these events is summarized in Figure 4C.

### Effects of U3 and U8 on nucleolar structure

As depletion of U3 or U8 triggers a potent nucleolar stress response, we wondered whether it might lead to loss of nucleolar integrity. So far, it has remained unclear whether activation of p53-dependent nucleolar surveillance is systematically accompanied by gross morphological alterations of the nucleolus.

**Figure 5:**
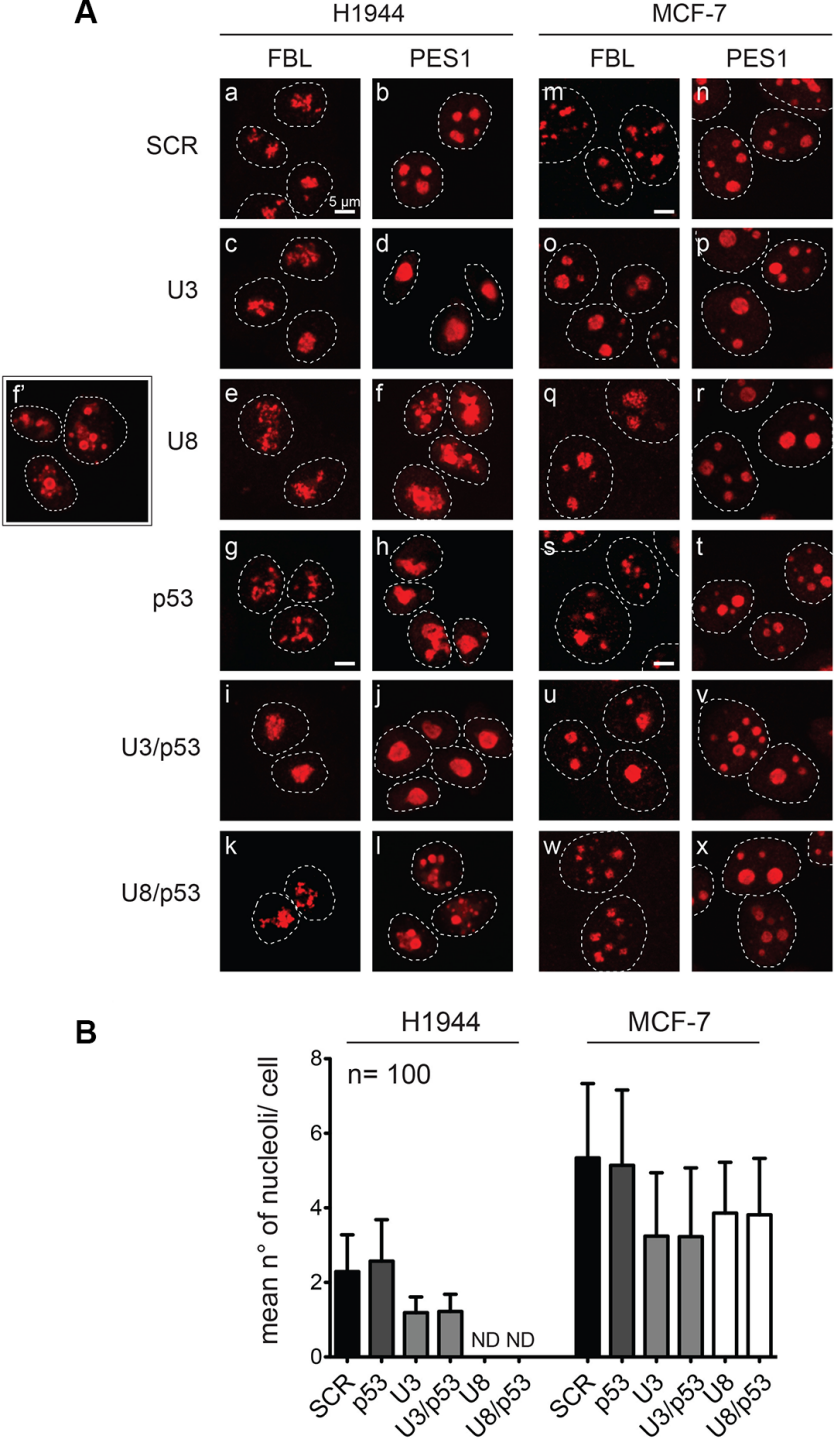
Effects of U3 and U8 depletion on nucleolar structure (**A**) H1944 and MCF-7 cells depleted for 2 days of U3, U8, p53, or a combination of p53 and U3 or U8 were processed for immunofluorescence and decorated with antibodies specific to the nucleolar proteins fibrillarin (FBL) or PES1 and imaged by spinning-disc confocal microscopy. As a control, cells were treated with a non-targeting silencer (SCR). The inset (panel f’) is showing additional cells treated in the same conditions as those shown in panel f. (**B**) The mean number of nucleoli was established by PES1 staining of 100 cells for each condition and plotted. In H1944 cells, upon U8 depletion, the number of nucleoli could not be determined (ND) owing to nucleolar disruption (see f, f’, and l, in panel A).

The nucleolus comprises three successive layers: the fibrillar center (FC), the dense fibrillar component (DFC), and the granular component (GC) [51]. H1944 and MCF-7 cells were depleted of U3 or U8 for 2 days, a sufficient time for the p53 steady-state level to be increased (see Figure 3A–3B). They were then subjected to immunofluorescence staining with antibodies against the DFC component fibrillarin (FBL) or the GC component PES1, and imaged by high-resolution spinning-disc confocal fluorescence microscopy (Figure 5).

Upon U3 depletion, the nucleolar structure was not grossly perturbed, but the nucleoli appeared larger, and the mean number of nucleoli per cell decreased by up to 50% (Figure 5A, compare panels d and b, o and m, p and n, and see Figure 5B for quantitation), interestingly, these are signs of terminally differentiating and senescent cells (see Discussion). U8 depletion had a similar effect on MCF-7 cells (Figure 5A, compare panels q and m, r and n, and see Figure 5B), but in H1944 cells it led to drastic nucleolar disruption, easily detectable by PES1 staining (Figure 5, compare panels f and b; panel f′ shows additional cells treated as in panel f). Strikingly, MCF-7 cells did not show this disruption (Figure 5, compare panels f–f′ and r). This effect, interestingly, is thus cell-specific.

Given the numerous connections between p53 and ribosome synthesis [47, 52], we next tested whether the effect of U3 or U8 depletion on nucleolar structure requires the presence of p53. We first noted that p53 depletion leads to mild disruption of nucleolar structure in H1944 cells (Figure 5, compare panels h and b), but interestingly not in MCF-7 cells (Figure 5, compare panels t and n). Although this effect is quite mild, this observation confirms that the nucleoli of H1944 cells are more sensitive to perturbations than those of MCF-7 cells. Apart from this observation, the effects of snoRNA depletion on nucleolar structure were highly similar in the presence and absence of p53 (Figure 5, compare panels i-l with c-f and u-x with o-r). The results of RT-qPCR and Western blotting showed that all the depletions were highly effective (Figure S6).

### The box C/D snoRNAs U3 and U8 are required for tumorigenesis *in vitro* and *in vivo*

**Figure 6:**
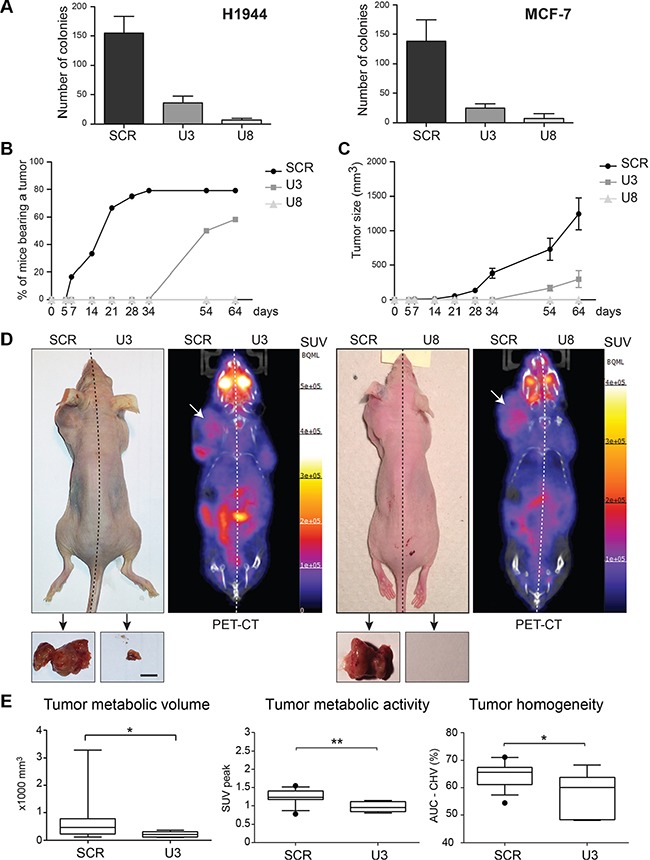
The box C/D snoRNAs U3 and U8 are required for *in vitro* and *in vivo* tumorigenesis (**A**) Soft-agar colony formation assay. H1944 and MCF-7 cells depleted of U3 or U8 for 3 days, and cells treated with a non-targeting control silencer (SCR), were layered on soft-agarose and incubated for 4-weeks, the colonies were stained with crystal blue and counted. Each data point represents a mean value of three independent experiments with SD. (**B–E**) Nude mouse xenograft experiment. Twelve nude mice were injected symmetrically in the upper body flanks with, on the right side of the animal, 5 million H1944 cells depleted of U3 or U8 for 3 days and, on the left side, 5 million H1944 cells treated with the non-targeting silencer control. (B) Percentages of mice bearing a tumor as monitored over a period of 64 days. The data show a one-month lag in tumor formation after injection of U3-suppressed cells, and no tumor formation after injection of U8-suppressed cells. (C) Tumor size estimated by caliper measurement. The data show that the tumors developed from U3-suppressed cells are substantially smaller and that no tumors developed from U8-suppressed cells. (D) One representative mouse (day 64) is shown for U3 depletion and one for U8 depletion. Large tumors are visible only in the flanks injected with control cancer cells (left side of the animals, white arrows). Left, digital photographic recording. Right, PET-CT tomograms. For simplicity, the ^18^F-FDG signals for the heart and bladder are not shown. The scale depicts the SUV (standard uptake values, see Materials and Methods) expressed in Bq/ml. Insets show resected tumors. Scale bar, 10 mm. (E) Tumor metabolic volume, tumor metabolic activity, and tumor heterogeneity were extracted and computed from the tomograms, according to refs [53, 75]. Tumor metabolic volume, tumor metabolic activity (through SUV peak), and tumor heterogeneity (through CSH-AUC) were compared between the U3-suppressed group and the control (scrambled depletion) group by means of an unpaired two-tailed *t* test, assuming equal variance between groups. The results, reported as *p*-values, were considered significant at *p* < 0.05. (*p ≤ 0.05; **p ≤ 0.01).

To test whether U3 and U8 affect *in vitro* tumorigenicity, we performed a colony formation assay on soft agar (Figure 6A). Cells depleted of U3 or U8 for three days and SCR-treated control cells were layered on noble agar, grown for a month with biweekly medium renewal, and colonies stained with crystal violet were counted. Upon U3 or U8 depletion, the capacity of H1944 and MCF-7 cells to form colonies was severely impaired (Figure 6A). The ability of U3-depleted cells to form colonies was reduced by approximately 75%, while the colony-forming ability of U8-depleted cells was almost totally abolished.

As U3 and U8 are important contributors to *in vitro* tumorigenicity, we next sought to determine whether they are also required for tumor formation *in vivo*. For this we used an ectopic xenograft mouse model (Materials and Methods and see [34]). Human lung cancer cells, depleted or not of U3 or U8, were implanted into the upper flanks of nude mice, and tumor evolution was monitored over a two-month period by palpation followed by caliper measurement (Figure 6B–6C) and by positron emission tomography (PET) combined with X-ray computed tomography (CT) imaging (Figure 6D–6E).

For each snoRNA, a cohort of twelve animals was used. Five million of H1944 cells depleted of U3 or U8 for 3 days were injected into one flank of each animal, and SCR-treated control cells were implanted symmetrically in the other flank. Analysis of the appearance of tumors as a function of time and according to the treatment applied to the injected cells revealed a one-month lag in the onset of tumorigenesis at sites injected with U3-depleted cells, as compared to sites injected with control cells (Figure 6B–6C). The tumors were also considerably smaller at the sites injected with U3-depleted cells (Figure 6C). At sites receiving U8-depleted cells, no tumor was detected at any time point of the experiment in any of the animals inspected (Figure 6B–6C).

PET imaging of ^18^F-fluorodeoxyglucose (^18^F-FDG) uptake was used to evaluate the metabolic activity of the tumors. To provide an anatomical correlation, X-ray CT scans were merged with the PET images (Figure 6D). Qualitative analysis of the PET images confirmed the drastically lower metabolic volumes of tumors derived from U3-suppressed versus control cells, and the absence of any tumor mass at sites injected with U8-suppressed cells (Figure 6D). Quantitative analysis of radiotracer accumulation within the tumor mass revealed the metabolically active part of the tumor mass (metabolic tumor volume), the maximum activity within the PET-based metabolic activity volume (SUVpeak), and the tumor heterogeneity (Figure 6E, [53]). The index of intratumoral heterogeneity was developed as a means of evaluating tumor responses to treatment [53]: increased tumor heterogeneity may reflect an increased ratio of necrotic versus cancerous tissue, a rather positive clinical parameter. Tumors derived from U3-suppressed cells showed a reduced metabolic volume, reduced metabolic activity, and increased heterogeneity as compared to tumors derived from control cells (Figure 6E). The increased heterogeneity of tumors derived from U3-suppressed cells indicates that the growth properties of these tumors are intrinsically different from those of aggressive control tumors. In conclusion, U3 is important in tumor evolution.

## DISCUSSION

Cell transformation results from disrupted regulation of cell growth. In order to proliferate, cells need to achieve a critical size, and for this they need to make sufficient amounts of proteins. This requires a sufficient number of actively translating ribosomes. In this work, we have focused on two conserved snoRNAs, the box C/D snoRNAs U3 and U8, whose functions in pre-rRNA processing have been characterized in various eukaryotic models, but surprisingly never in humans (see Introduction). Our aim was to see what role these snoRNAs might play in ribosome biogenesis, nucleolar structure, the nucleolar stress response, and tumorigenesis.

Our data obtained with breast, cervix, colon, and lung cancer cells demonstrate that U3 and U8 are required, respectively, for early and late pre-rRNA processing steps (Figures 1 and 2). We show that U3-depleted cells are impaired in synthesis of the small ribosomal subunit, while large subunit production is inhibited in U8-depleted cells (Figure 1). We reveal that in breast and lung tumor cells, U3 or U8 depletion triggers a particularly powerful p53-dependent antitumor surveillance response leading to p53 stabilization, cell cycle arrest, and apoptosis (Figures 3 and 4). We further show that U3 depletion strongly inhibits, and U8 depletion almost totally abolishes the *in vitro* tumorigenicity of breast and lung cancer cells (Figure 6). We provide *in vivo* confirmation of this finding, demonstrating in a mouse xenograft model that U3- and U8-suppressed lung cancer cells have a diminished or abolished tumor-forming capacity (Figure 6).

Associations have recently been reported between snoRNAs and tumorigenesis (see Introduction), but in most cases the precise involvement of snoRNAs in cell transformation is not known. Given the known functions of snoRNAs in ribosome biogenesis, i.e. their action as either antisense guides targeting specific nucleotides for post-transcriptional modification or as *trans*-acting factors required for pre-rRNA processing (as shown here for U3 and U8), their involvement in tumorigenesis is likely linked mostly to impaired ribosome production and the resulting translational deficiencies. This particularly concerns snoRNAs involved in rRNA modification, as some of them display tissue-specific expression. According to the tissue, specialized ribosomes may be produced, with specific rRNA modification patterns conferring differential translational capabilities ([39, 54], reviewed in [6]). The ‘choice’ to initiate translation at cap-dependent sites or internal *r*ibosomal *e*ntry *s*ites (IRES) is notably influenced by specific rRNA modification patterns [39, 55, 56]. This is directly relevant to tumorigenesis, as transcripts of several tumor-suppressor genes and proto-oncogenes rely specifically on IRES initiation to be translated [57]. As for U3 and U8, since they are essential to pre-rRNA processing and subunit biogenesis, a straightforward hypothesis is that suppression of tumorigenesis upon their depletion is due to reduced ribosome synthesis, concomitant activation of nucleolar stress, and the resulting remarkable stabilization of p53.

According to the current model of nucleolar surveillance activation, disruption of ribosome biogenesis leads to accumulation of unassembled ribosomal components, including the r-proteins uL5 and uL18. These two proteins associate with the 5S rRNA to form a 5S RNP, which titrates the ubiquitin ligase Hdm2 [46]. When Hdm2 is sequestered, p53 is no longer ubiquitinated and degraded by the proteasome. Its accumulation triggers expression of pro-apoptotic genes leading to cell death. In keeping with this model, we show that the effect of snoRNA depletion on the steady-state level of p53 depends strictly on the presence of uL5 and uL18 (Figure 3). In the case of U8 depletion, the nucleolar stress response is particularly pronounced, with up to a 25-fold increase in the p53 level (Figure 3). This dramatic response likely explains why U8 depletion almost totally abolishes the *in vitro* tumorigenicity of cancer cells and why our cohort of mice injected with U8-suppressed cancer cells failed to develop any tumor.

Interestingly, we find that U8 depletion, in striking contrast to U3 depletion, diminishes the amounts of uL5 and uL18 (Figure 3). As discussed above, uL5 and uL18 form with the 5S rRNA the 5S RNP, which constitutes the central protuberance (CP), a prominent landmark on mature 60S subunits [58]. Final integration of the CP into 60S subunit precursors is a late assembly event in the pathway of large subunit maturation ([59, 60]). U8 is active in late pre-rRNA processing steps required for the synthesis of the large subunit, in contrast to U3, involved in early processing reactions important in small subunit biogenesis (Figures 1–2). We note that the intracellular pool of 5S rRNA is reduced after U8 depletion, but not after U3 depletion (Figure 1). We speculate that this may explain why U8 is required for normal uL5 and uL18 accumulation. We also find that uL5 and uL18 are required for each other's metabolic stability (Figure 3). We suggest that this also reflects the presence of uL5 and uL18 within a trimeric 5S RNP.

The nucleolus is a powerful indicator of the health status of a cell [61]. Accordingly, cancer cells frequently display more numerous, larger, and deformed nucleoli. We have made two significant observations regarding overall nucleolar structure in U3- and U8-depleted cancer cells. Firstly, after U8 or p53 depletion, we find the nucleoli of H1944 lung cancer cells to be more prone to disruption than those of MCF-7 breast cancer cells (Figure 5). Importantly, as p53 was stabilized in both the H1944 and MCF-7 cell lines, this shows that activation of nucleolar stress does not rely on gross alteration of nucleolar structure. Secondly, we find the nucleoli of U3-depleted H1944 cells and those of U3- or U8-depleted MCF-7 cells to be larger and less numerous (Figure 5). Typically, this is the consequence of nucleolar fusion, which has been linked, strikingly, to cell senescence and tumorigenesis in a three-dimensional mammary epithelial cell culture model that recapitulates the early stages of breast cancer [62]. Directly relevant to our interpretation of nucleolar fusion as a sign of cell senescence is the recent observation that the 5S RNP-mediated nucleolar stress response can act as a senescence inducer under conditions of oncogenic or replicative stress [63].

In agreement with reduced ribosome biogenesis, tumors derived from U3-suppressed cells display a markedly reduced tumor metabolic volume and reduced metabolic activity (Figure 6). Remarkably, these tumors also show increased heterogeneity in the uptake of the FDG metabolic tracer, indicating distinct growth properties. In addition to their functions in ribosome biogenesis, snoRNAs and stable fragments derived from them have recently been attributed non-conventional functions that may be relevant to their involvement in tumorigenesis. SNORD50A and SNORD50B, for example, have been shown to bind directly to K-Ras, inhibiting its function [41]. SnoRNA-derived RNAs (sdRNAs) have been attributed regulatory functions as interfering antisense RNAs in alternative splicing regulation [64], mRNA translation repression, and mRNA turnover ([65, 66], discussed in [67–69]). While no such functions have been directly attributed to U3 or U8 thus far, their high abundance and metabolic stability in cells suggest that they may exert such non-conventional functions. We speculate that the increased tumor heterogeneity observed after U3 depletion might reflect the loss of such functions. Our model is supported by the recent observation that multiple sdRNAs are produced from both U3 and U8 in prostate cancer [70].

In a recent study, Su *et al.* showed that the tumorigenicity of cancer cells can be reduced *in vitro* and *in vivo* after depletion of proteins important for the metabolic stability of all box C/D snoRNAs, and that this involves p53-dependent cell cycle arrest [30]. Our results on U3 and U8 are fully compatible with theirs and also extend them considerably. By targeting for depletion either a protein shared by all box C/D snoRNPs (such as the methyltransferase fibrillarin) or an assembly factor required for their packaging into functional ribonucleoprotein complexes, Su *et al.* eliminated without distinction an entire family of several hundred small nucleolar RNAs [30]. This made it impossible to distinguish whether the observed effects on tumorigenicity (and p53 nucleolar stress activation) were due to loss of the myriad snoRNAs involved in pre-rRNA 2′-*O* methylation, to loss of snoRNAs involved in pre-rRNA processing (such as U3 or U8), or to both. Furthermore, as these authors did not address effects on nucleolar structure or on any other aspect of ribosome biogenesis (such as pre-rRNA processing or 2′-*O* methylation), the molecular basis of their findings remains unclear. In the present study, in contrast, we have chosen to deplete cells of a single snoRNA family member, either U3 or U8. We can thus conclude beyond a doubt that the effects we observe are due to severe inhibition of pre-rRNA processing events and to impairment of ribosomal subunit production.

Hyperactive ribosomal biogenesis is a common feature of cancer cells, which appear more sensitive than non-cancerous cells to inhibition of ribosome synthesis [71, 72]. Previous work has focused mainly on the synthesis of ribosomal RNAs [71, 72]. We have targeted, instead, a post-transcriptional step: pre-rRNA processing. We find that using an antisense silencer to deplete cells of a single small non-coding RNA molecule (U3 or U8) is sufficient to block ribosome biogenesis and to elicit a potent p53-dependent anti-tumor surveillance response. This has important implications in cancer research, as tumors are well known to depend on their ability to reinforce their ribosome synthesis capacity to ensure rapid cell division. Preventing this reinforcement by inhibiting pre-rRNA processing might thus be a good way to impair tumor development. As discussed above, several r-proteins are well known to play an important role in regulating the p53 steady-state level. In a recent work, each of the eighty human ribosomal proteins was depleted, one by one, and tested for its exact involvement in p53 homeostasis [48]. Depletion of the strongest contributors led to a 5- to 10-fold increase in the steady state accumulation of p53 [48]. By comparison, depletion of U3 or U8 leads to 15- to 25-fold p53 stabilization. This is a considerable p53 increase, which makes us confident that silencing a single snoRNA essential to pre-rRNA processing is an avenue well worth exploring with a view to developing novel anticancer strategies.

## MATERIALS AND METHODS

### Cell culture and growth curves

Human cells were grown at 37°C under 5% CO_2._ Growth curves were determined by direct cell counting with a Scepter (EMD Millipore, Billerica, MA, USA). All cell lines used in this work (Table S1) were obtained directly from ATCC and passaged in the laboratory for fewer than 6 months after receipt. All cell lines were diagnosed by short tandem repeat (STR) profiling by ATCC. For NCI-H1944 (CRL-5907), the cell lot number was 61487231; for MCF7 (HTB-22) it was 61235352.

### Cell viability assays

Cell viability assays were carried out with a Muse cell analyzer (EMD Millipore) and a cell count and viability assay kit (Millipore, MCH100102), according to the manufacturer's instructions.

### Cell cycle and apoptosis analysis

DNA content (cell cycle), and annexin V/7-AAD expression (apoptosis) were established with a Muse cell analyzer (EMD Millipore) and dedicated kits (EMD Millipore, MCH100106 and MCH100105).

### Gene expression perturbation methods

Human cells were reverse transfected as follows: 40 μM ASO (Integrated DNA Technologies, Coralville, IA, USA) or 20 μM Silencer select^®^ siRNA (Thermo Fisher Scientific, Waltham, MA, USA) and 4 μL of Lipofectamine RNAiMAX (Thermo Fisher Scientific) were mixed with 500 μL Opti-MEM (Thermo Fisher Scientific) in each well of a 6-well plate. After a 20-minute incubation at room temperature (RT), 3 × 10^5^ cells resuspended in 2.5 mL antibiotic-free medium were seeded into each well. Inactivation was carried out for the desired time. In the immunofluorescence experiments, depletions were performed in 96-well plates (Porvair Sciences, Leatherhead, UK). A transfection reagent mix (0.125 μl Interferin (Polyplus-transfection, Illkirch, France) and 20 μl Optimem (Thermo Fisher Scientific)) was added to each plate well and left to set for 10 min at RT. A specific ASO (10 μl of a 200 nM stock) or siRNA (10 μl of a 100 nM stock) was added to the mix and left to set for another 30 min at RT. Cells (70 μl of a suspension containing 200,000 cells/ml) were added to each well and the plates incubated for 2 days. All ASOs and siRNAs used in this study are listed in Tables S2 and S3. The efficiency of snoRNA depletion was validated by RT-qPCR as described in ref. [73], except that we used amplicons for human U6 snRNA as an endogenous control. The sequences of the primers used are listed in Table S4. Data were analyzed with the StepOne software (v 2.1) (Thermo Fisher Scientific) and the comparative threshold cycle (CT) method (“Livak” method) was used for quantification.

### Protein biochemistry

Total protein extraction and Western blotting were performed exactly as described in ref. [73]. The antibodies used in this study are listed in Table S5.

### Polysome profile analysis

Polysome profile analysis was performed as described in ref. [73].

### Pre-rRNA processing analysis

Total RNA extraction, quantification, gel electrophoresis, Northern blotting, and RNA quantification were performed as described in ref. [73]. The probes used are described in Table S4.

### Immunofluorescence analysis

After 2 days of ASO-mediated depletion, cells were fixed in 4% formaldehyde, washed in PBS, and blocked in PBS supplemented with 5% BSA, 0.3% Triton X-100 for 1 hour at RT. The cells were incubated with a primary antibody overnight at 4°C, washed in PBS, and incubated with a secondary antibody coupled to Alexa Fluor 594 (Thermo Fisher Scientific, 1:1,000) for 1 hour at RT. The primary antibody used was either an anti-FBL (Antibodies online, Atlanta, GA, USA, ABIN361375, 1:250) or an anti-PES1 (Ascension GmbH, 1:500). Finally, the cells were stained with DAPI (Sigma-Aldrich). Imaging was performed on an Axio Observer Z1 (Zeiss, Oberkochen, Germany) driven by MetaMorph (MDS Analytical Technologies, Sunnyvale, CA, USA). Images were captured in confocal mode using a Yokogawa (Musashino, Japan) spinning disk head and the Coolsnap HQ2 camera with laser lines from Roper (Sarasota, FL, USA) (405 nm 100 mW Vortran and 561 nm 50 mW Cobolt Jive) and a Zeiss EC Plan-NeoFluar 40x/0,75 Ph2 objective.

### *In vitro* tumorigenesis assay

Colony formation assays were performed exactly as previously described [34].

### *In vivo* xenograft model

All animal studies were performed in compliance with the European Ethics Committee guidelines. The study protocol was approved by the local Experimental Animal Ethics Committee of the BUC-CMMI, ref. CMMI-2013-05. In practice, twenty-four 6–8-week-old female nude mice were injected subcutaneously in the upper left flank with 5 million H1944 human non-small cell lung carcinoma cells transfected with a negative non-targeting control ASO (SCR), and in the upper right flank with the same number of cells depleted of U3 (12 mice) or U8 (12 mice) for 3 days. During the intervention, the mice were anesthetized under gaseous anesthesia (4% isoflurane evaporated by an O_2_ flow at 3l/min for induction and 1.5–2% isoflurane evaporated by an O_2_ flow at 1.5-2l/min for maintenance). The mice were weighed once a week before PET-CT imaging. Tumor size was monitored once a week by palpation followed by caliper measurement.

### ^18^F-FDG PET-CT imaging and image processing for quantitative analysis

PET-CT imaging of the ^18^F-FDG signal was performed to assess the metabolic activity of the tumor mass. Mice were imaged after 8, 15, 22, 29, 58, and 65 days after injection of the tumor cells. The day before imaging, the mice were fasted overnight. They were injected intravenously (lateral tail vein) with 3.9 MBq to 5.5 MBq of ^18^F-FDG synthesized at the PET/Biomedical Cyclotron Unit of the Nuclear Medicine Department at ULB-Hôpital Érasme and kept under 2.5%-isoflurane anesthesia for 10 minutes post-injection to limit tracer uptake within skeletal muscles and brown adipose tissue ([74]). PET imaging was performed 60 min after ^18^F-FDG injection for 15 min under isoflurane anesthesia; this was done with a μPET-μCT scanner (nanoPET-CT, Mediso, Budapest, Hungary) in 3-to-1 coincidence mode. PET acquisition was followed by CT acquisition (55 kV, 145 μA, 1100 ms per projection, 180 projections per rotation, pitch of 1, a frame binning of 4 by 4, and a cubic reconstructed voxel size of 284 μm). All PET images were also corrected for random counts, dead time, and decay. The PET acquisitions were reconstructed by means of a fully 3-dimensional iterative OSEM reconstruction algorithm (4 iterations, 6 subsets, intermediate regularization setting, median filtering period defined from iteration counts). CT images were used to obtain attenuation-corrected and scatter-corrected PET images. PET-CT analyses were performed with Vivoquant1.23 (inviCRO, Boston, USA). A three-dimensional region of interest (ROI) was drawn on the CT-based tumor mass and used as input to define a PET-based ROI corresponding to the metabolic activity volume of the tumor. Segmentation of this PET-based ROI was performed using a thresholding method, with a threshold set at 30% of the maximum activity value within the tumor ROI. The PET was then smoothed with a 3D Gaussian filter with a full width at tenth maximum (FWTM) of 1.2 mm, corresponding roughly to 3 voxels, to reduce noise bias. Maximum activity within the smoothed PET-based tumor ROI was expressed in Bq/mL and was subsequently divided by the ratio of A_0_ (decay-corrected injected activity at the start of the PET acquisition, in Bq) to animal weight (in g). This normalized maximum value is called Peak Standard Uptake Value, or SUVpeak, by analogy to hospital practice. The heterogeneity of tracer uptake was quantified with a metric derived from cumulative SUV-volume histograms (CSHs) [75]. The CSHs are obtained by plotting the percent volume of the delineated tumor with a SUV above a certain threshold, for thresholds ranging from 0 to 100% of SUVpeak. The area under the CSH curve (AUC) is then computed and used as a quantitative index of tracer uptake heterogeneity (lower values correspond to higher heterogeneity) [53].

## SUPPLEMENTARY MATERIALS FIGURES AND TABLES


