# Supplementary material for: The human box C/D snoRNAs U3 and U8 are required for pre-rRNA processing and tumorigenesis
Source: Oncotarget. 2016 Aug 9;7(37):59519–34. doi: 10.18632/oncotarget.11148 (PMC5312328; doi:10.18632/oncotarget.11148)
Supplement: Supplementary file 1 [file oncotarget-07-59519-s001.pdf]

The human box C/D snoRNAs U3 and U8 are required for pre-rRNA processing and tumorigenesis

Supplementary Materials

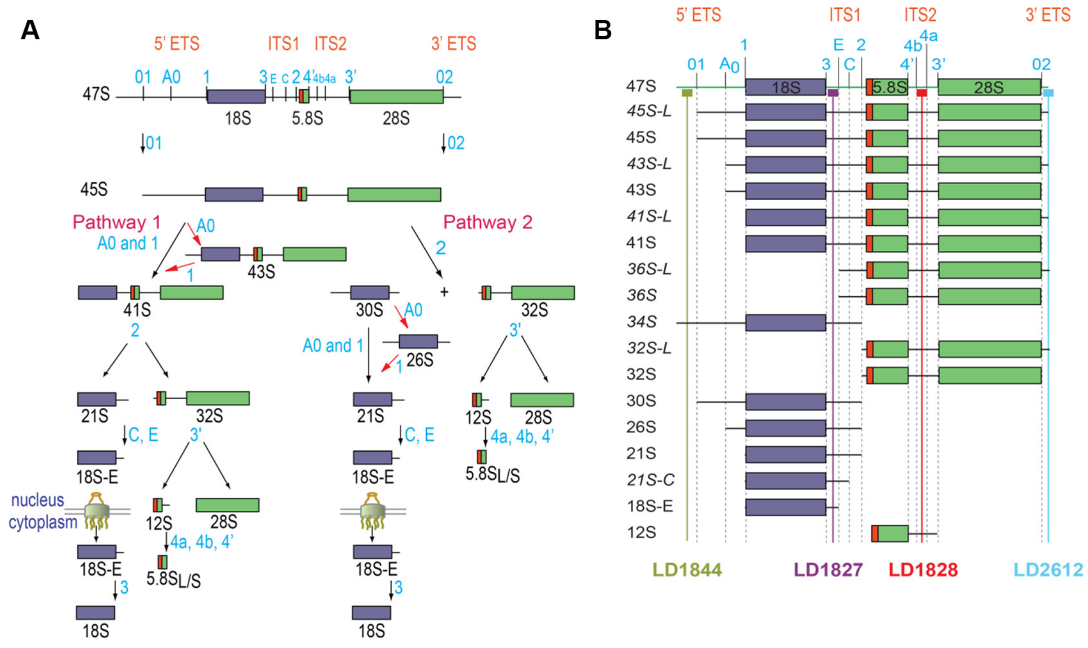

Supplementary Figure S1: Pre-rRNA processing pathway in human cells and major pre-rRNA intermediates. (**A**) Three of the four mature rRNAs, the 18S, 5.8S, and 28S rRNAs are produced from a single RNA Pol I transcript (47S). The 18S rRNA is the RNA component of the small subunit (40S); 5.8S and 28S are incorporated into the large subunit (60S). There is a third rRNA in the 60S subunit, 5S, which is independently produced by RNA Pol III (not shown). The mature sequences are embedded in noncoding 5′ and 3′ external transcribed spacers (ETS) and internal transcribed spacers (ITS1 and 2). Cleavage sites (in cyan) and alternative pathways are indicated. For details, see Www.RibosomeSynthesis.Com. (**B**) Northern blot probes used in this work (LD1844, LD1827, LD1828, and LD2612) highlighting the pre-rRNA species detected (see Table S4).

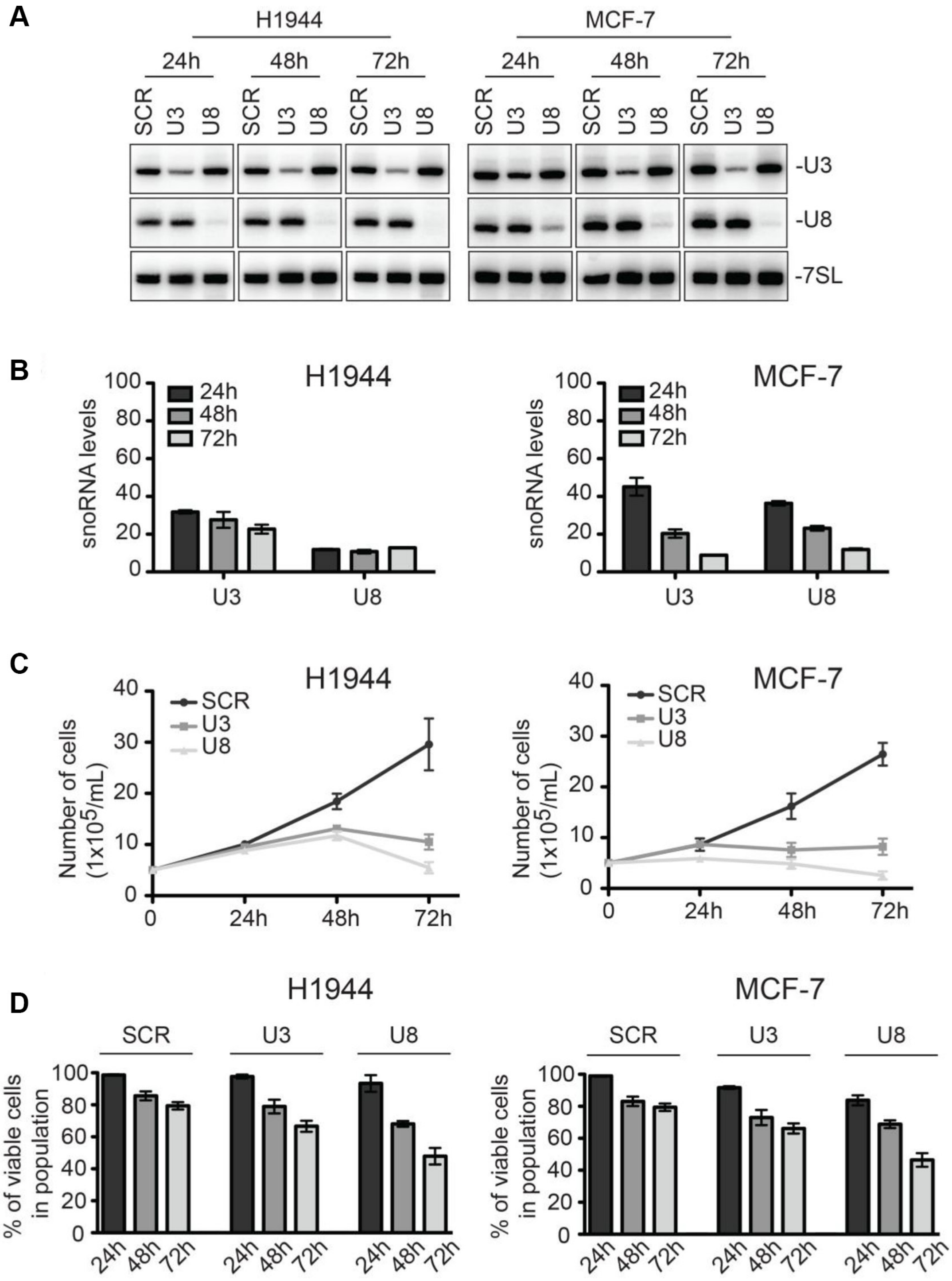

Supplementary Figure S2: Efficency of ASO-mediated snoRNA depletion. To efficiently deplete the abundant and stable snoRNAs, we used phosphorothioate-modified chimeric antisense oligonucleotides (ASO, see materials and methods and Table S2). (**A**) Northern-blot analysis. Total RNA extracted from H1944 or MCF-7 cells depleted of U3, or U8, for 1-, 2-, or 3-days was analyzed by Northern blotting with specific probes. As a control, cells were treated with a non-targeting control (SCR) silencer. 7SL probing was used as a loading control. (**B**) Quantification by phosphorimager analysis of the signals shown in panel A. snoRNA levels are expressed as percentages of the level in SCR-treated cells. The mean of three independent experiments is shown (biological triplicates). In H1944 cells, depletion was consistently more efficient for U8 than for U3, leading respectively to ~10% and ~25–30% residual snoRNAs after 3 days of depletion. In MCF-7 cells, depletions was more gradual and reached ~10–15% residual snoRNAs after 3 days for both U3 and U8. (**C**) The total number of cells after snoRNA depletion was established by coulter counting with a Scepter. (**D**) The number of viable cells after snoRNA depletion was determined by staining with a vital dye and counting with a Muse.

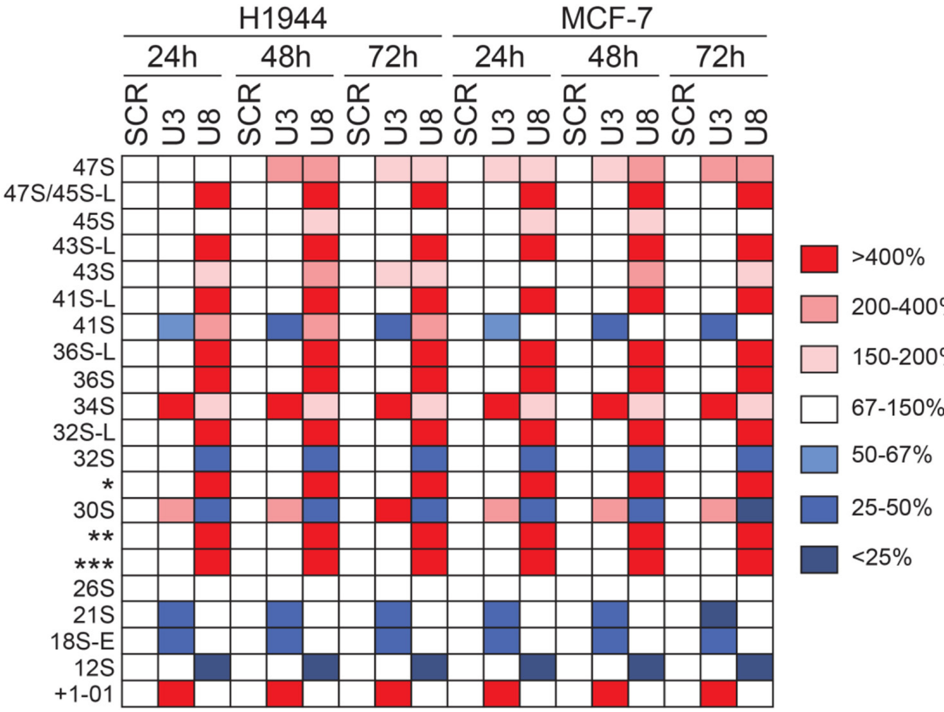

Supplementary Figure S3: Quantification of the Northern blot signals shown in Figure 2. The bands detected in the Northern blot analysis of Figure 2 were quantitated with a Phosphorimager (FLA-7000, Fujifilm) and their intensity expressed in a heatmap as a percentage of the signal observed in control cells (SCR).

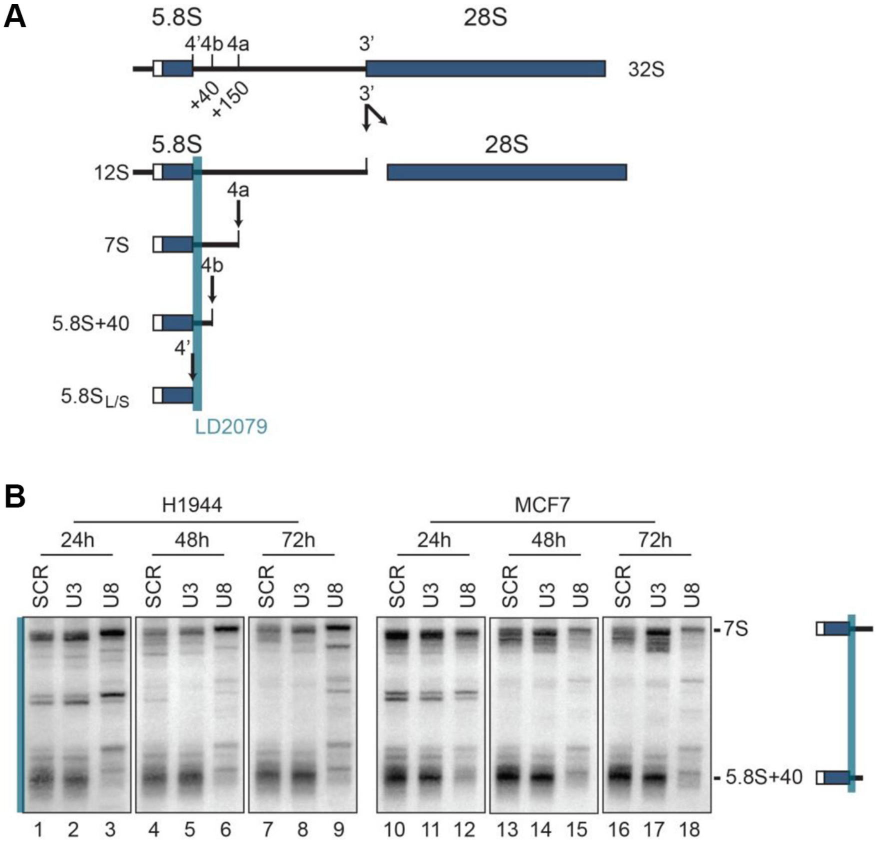

Supplementary Figure S4: Northern blot analysis of 12S pre-rRNA maturation. (**A**) Maturation pathway of 5.8S rRNA. The probe LD2079 used in panel B is highlighted. (**B**) The total RNA samples presented in Figure 2 were resolved on denaturing acrylamide gels for high-resolution analysis and tested by Northern blotting with probe LD2079.

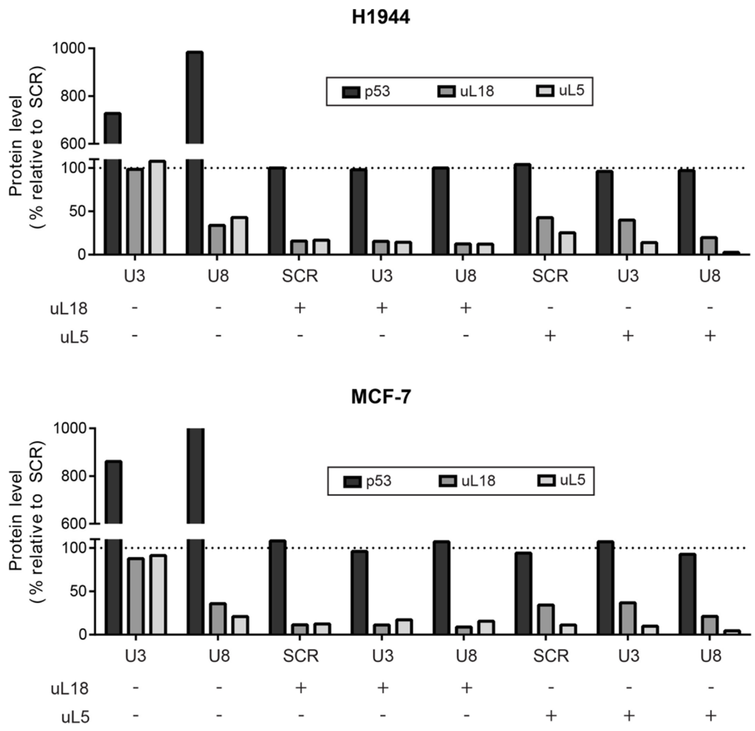

Supplementary Figure S5: Quantification of Western blot signals shown in Figure 3B. Each band was quantitated with a Chemidoc (BioRad).

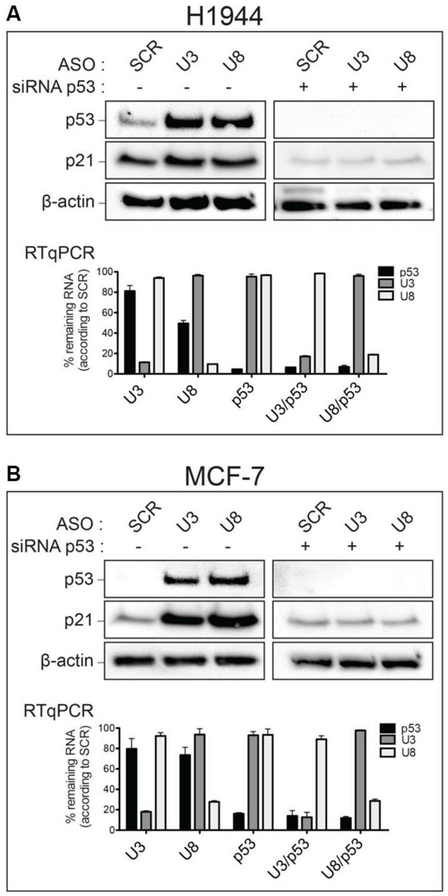

Supplementary Figure S6: Efficiency of the snoRNA and protein depletions shown in Figure 5. The depletion efficiencies were established by Western blotting (upper panels) and by RT-qPCR (lower panels) for the experiments conducted in H1944 (panel A) and MCF-7 cells (panel B).

Supplementary Table S1: Human cell lines

Name	Media	Source
H1944	RPMI-1640 (ATCC)	ATCC
MCF-7	Eagle's minimum essential medium (ATCC)	ATCC
H1975	RPMI-1640 (ATCC)	ATCC
A549	F-12K (ATCC)	ATCC
DMS-53	DMEM (Sigma)	ATCC
BT-549	RPMI-1640 (ATCC)	ATCC
HeLa	DMEM (Sigma)	ATCC
HCT116 +/+	McCoy's 5a (ATCC)	ATCC
HCT116 −/−	McCoy's 5a (ATCC)	ATCC

All cell lines were purchased from the ATCC repository and regularly tested for contamination with the LookOut mycoplasma PCR detection kit (Sigma-Aldrich, MP0035). All media were supplemented with 50 U/mL penicillin/50 mg/mL streptomycin (Life Technologies) and 10% fetal bovine serum (A & E scientific).

Supplementary Table S2: ASO silencers

SCR	mUmCmAmCm*CTTCACCCTCT*mCmCmAmCmU
U3	mUmUmCmGm*GTGCTCTACAC*mGmUmUmCmA
U8	mGmGmAmUm*UATCCCACCTG*mAmCmGmAmU

N and mN are deoxynucleotide and 2′-*O*-methoxyethylribonucleotide, respectively. Phosphodiester backbones are phosphorothioates.

Supplementary Table S3: siRNAs

Target	Sequence
p53	GAAAUUUGCGUGUGGAGUAtt
RPL5	GACGAGAGGGUAAAACUGAtt
RPL11	GGUGCGGGAGUAUGAGUUAtt

Supplementary Table S4: Oligonucleotides used in Northern blotting and RT-qPCR

Northern-blot:

LD1827	CCTCGCCCTCCGGGCTCCGTTAATGATC	ITS1
LD1828	CTGCGAGGGAACCCCCAGCCGCGCA	ITS2
LD1844	CGGAGGCCCAACCTCTCCGACGACAGGTCGCCAGAGGACAGCGTG	5′-ETS
LD2079	GGGGCGATTGATCGGCAAGCGACGCTC	ITS2
LD2133	GCTCCGTTTCCGACCTGGGCC	7SL
LD2134	GCTCTACACGTTCAGAGAAACTTCTCTAGTAACACACTATAGAAATGATCC	U3
LD2136	GTTCTAATCTGCCCTCCGGAGGAGGAACAG	U8
LD2612	GAGGAGGCGGGAACCGAAGAAGCGG	3′-ETS

RT-qPCR:

LD2650	GCGCACAGAGGAAGAGAATC	p53 (Fwd)
LD2651	CAAGGCCTCATTCAGCTCTC	p53 (Rev)
LD3238	CGTCAGGTGGGATAATCCTT	U8 (Fwd)
LD3239	GGGTGTTGCAAGTCCTGATT	U8 (Rev)
LD3240	GCTTCGGCAGCACATATACTAA	U6 (Fwd)
LD3241	CGCTTCACGAATTTGCGTGTCAT	U6 (Rev)
LD3270	CGTGTAGAGCACCGAAAACC	U3 (Fwd)
LD3271	CACTCAGACCGCGTTCTCTC	U3 (Rev)

Supplementary Table S5: Antibodies used in Western blotting

Target	Dilution	Source
p53	1: 2000	Bethyl Laboratories (A300-247A)
p21	1:2000	Cell Signaling (#2947)
RPL11	1:4000	Bethyl Laboratories (A303-931A)
RPL5	1:4000	Bethyl Laboratories (A303-9331)
β-actin	1:5000	Santa Cruz Biotechnology (sc-130301)
